# Molecular Mechanisms of Amyloid-β Self-Assembly Seeded by In Vivo-Derived Fibrils and Inhibitory Effects of the BRICHOS Chaperone

**DOI:** 10.1021/acschemneuro.3c00044

**Published:** 2023-04-06

**Authors:** Rakesh Kumar, Luis Enrique Arroyo-García, Shaffi Manchanda, Laurène Adam, Giusy Pizzirusso, Henrik Biverstål, Per Nilsson, André Fisahn, Jan Johansson, Axel Abelein

**Affiliations:** † Department of Biosciences and Nutrition, Karolinska Institutet, 141 52 Huddinge, Sweden; ‡ Division of Neurogeriatrics; Center for Alzheimer Research; Department of Neurobiology, Care Sciences and Society, Karolinska Institutet, 171 64 Solna, Sweden; § Department of Women’s and Children’s Health, Karolinska Institutet, 171 64 Solna, Sweden

**Keywords:** Aggregation kinetics, App knock-in Alzheimer’s disease mouse models, amyloid-β peptide, in vivo-derived fibrils

## Abstract

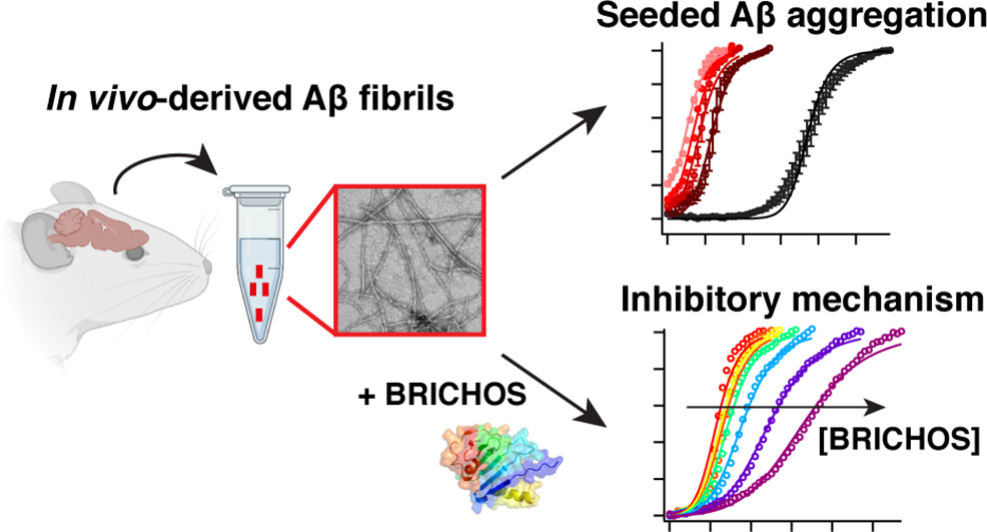

Self-replication of amyloid-β-peptide (Aβ) fibril formation is a hallmark in Alzheimer’s disease (AD). Detailed insights have been obtained in Aβ self-assembly in vitro, yet whether similar mechanisms are relevant in vivo has remained elusive. Here, we investigated the ability of in vivo-derived Aβ fibrils from two different amyloid precursor protein knock-in AD mouse models to seed Aβ42 aggregation, where we quantified the microscopic rate constants. We found that the nucleation mechanism of in vivo-derived fibril-seeded Aβ42 aggregation can be described with the same kinetic model as that in vitro. Further, we identified the inhibitory mechanism of the anti-amyloid BRICHOS chaperone on seeded Aβ42 fibrillization, revealing a suppression of secondary nucleation and fibril elongation, which is strikingly similar as observed in vitro. These findings hence provide a molecular understanding of the Aβ42 nucleation process triggered by in vivo-derived Aβ42 propagons, providing a framework for the search for new AD therapeutics.

## Introduction

Aggregation of proteins into insoluble amyloid fibrils is a common phenomenon observed in various neurodegenerative disorders, such as Alzheimer’s and Parkinson’s disease.^1,2^ In Alzheimer’s disease (AD), the most prevalent neurodegenerative disease, 40 or 42 residue long amyloid-β peptides (Aβ) self-assemble into amyloid fibrils, which are deposited as extracellular plaques in the brains of AD patients.^1^ Accumulating evidence supports that the self-assembly process is linked to the progression of pathology and cognitive decline in AD.^2^ To understand the molecular mechanism of Aβ aggregation, the aggregation kinetics of Aβ40 and Aβ42 has been studied in great detail in vitro.^3−7^ In particular, developing different nucleation models has provided valuable insights into the molecular processes of protein self-assembly,^8,9^ which were exemplified to study Aβ aggregation.^3−7^ In these kinetic models, the molecular self-assembly is determined by a set of microscopic nucleation rate constants related to primary and secondary nucleation as well as fibril-end elongation.^8,9^ First nucleation units are formed by primary nucleation, which further grow into amyloid fibrils. Processes related to the secondary products of the kinetic reaction—the amyloid fibrils—are referred to as secondary nucleation. Here, the surface of fibrils can catalyze the formation of new nucleation units, which subsequently can convert to oligomeric assemblies. Aggregation reactions that are accelerated by preformed fibrils are dominated by secondary nucleation processes, such as Aβ40 and Aβ42 aggregation under various in vitro conditions such as low^3,4,7^ and high salt^5,6^ or in the presence of cerebrospinal fluid.^10^ However, the proliferation and aggregation mechanism of Aβ in vivo has not been established.

To model AD, various amyloid precursor protein (App) transgenic mouse lines have been used.^11^ One major drawback with these previously generated AD mouse models is the overexpression of full-length App rather than specifically producing Aβ, which creates artifacts as overexpressed App and non-Aβ processing products affect various signaling pathways in the cell.^11,12^ To overcome these limitations, we used App knock-in mouse models with humanized Aβ42 sequence.^12^ The App^NL-F^ model harbors both the Swedish and Beyreuther/Iberian mutations, whereas the App^NL-G-F^ model additionally includes the Artic mutation. The Artic (Aβ E22G) mutation is located within the Aβ42 sequence, while the other mutations are located either upstream (Swedish) or downstream (Beyreuther and Iberian) of the Aβ42 sequence.^12^ The Swedish mutation increases the total amount of Aβ production by increasing the level of β-secretase cleavage, while the Beyreuther/Iberian mutation shifts the ratio of Aβ42/Aβ40 to the more neurotoxic Aβ42 species.^12^ The addition of the Artic mutation in the App^NL-G-F^ model introduces aggressive amyloidosis already at an early age, and severe memory impairment is observed around three times faster than for the App^NL-F^ model.^12^ A recent study showed that the molecular structure of App^NL-F^ fibrils largely coincides with human brain type II filaments,^13^ supporting the relevance of this mouse model.

Molecular chaperones are parts of the cellular protein control machinery assisting in, among others, protein synthesis, folding, and degradation.^14^ More recently, molecular chaperones or chaperone-like proteins have also been shown to suppress the amyloid formation of various amyloidogenic proteins.^15−19,42^ Such amyloid-suppressing chaperones comprise examples from the small heat shock protein (HSP) family, extracellular chaperones, and members of the BRICHOS domain family.^15−19,42^ A promising candidate for amyloid-modulating therapeutic strategies is BRICHOS from Bri2, since it is expressed in the human brain,^20^ passes the blood-brain barrier in mice,^21^ and inhibits Aβ aggregation and prevents Aβ-associated toxicity in vitro.^22,23^ Importantly, a recent study wherein App^NL-F^ and App^NL-G-F^ mice were intravenously treated with recombinant human (rh) Bri2 BRICHOS showed reduced neuroinflammation and improved working memory and object recognition.^24,42^

Whether the molecular mechanism of Aβ aggregation accelerated by in vivo-derived Aβ42 fibrils is the same as that for in vitro fibrils is still an open question. In addition, further research is demanded on whether the inhibitory mechanism of BRICHOS on Aβ42 self-assembly determined in vitro can be linked to the observed effects in the treatment studies of AD mice.

In this study, we demonstrate that Aβ42 fibrils extracted from App^NL-F^ and App^NL-G-F^ mice exhibited similar fibrillar morphology and fibril diameters and caused similar impact on hippocampal γ-oscillations ex vivo. These in vivo-derived Aβ42 fibrils efficiently seeded the aggregation of recombinant Aβ42 monomers, where the aggregation kinetics could be fitted to a nucleation model, thus, revealing the individual contributions of the microscopic events modulated by the presence of in vivo-derived fibrils. Notably, rh Bri2 BRICHOS has the ability to suppress in vivo-fibril-seeded Aβ42 self-replication by selectively inhibiting nucleation events linked to secondary nucleation and fibril-end elongation. These findings provide detailed knowledge of the molecular inhibitory mechanism of Aβ42 fibrillization by rh Bri2 BRICHOS in the presence of in vivo-derived fibrils.

## Results and Discussion

### Aβ Fibril Extraction from App^NL-F^ and App^NL-G-F^ Mouse Brain

**Figure 1:**
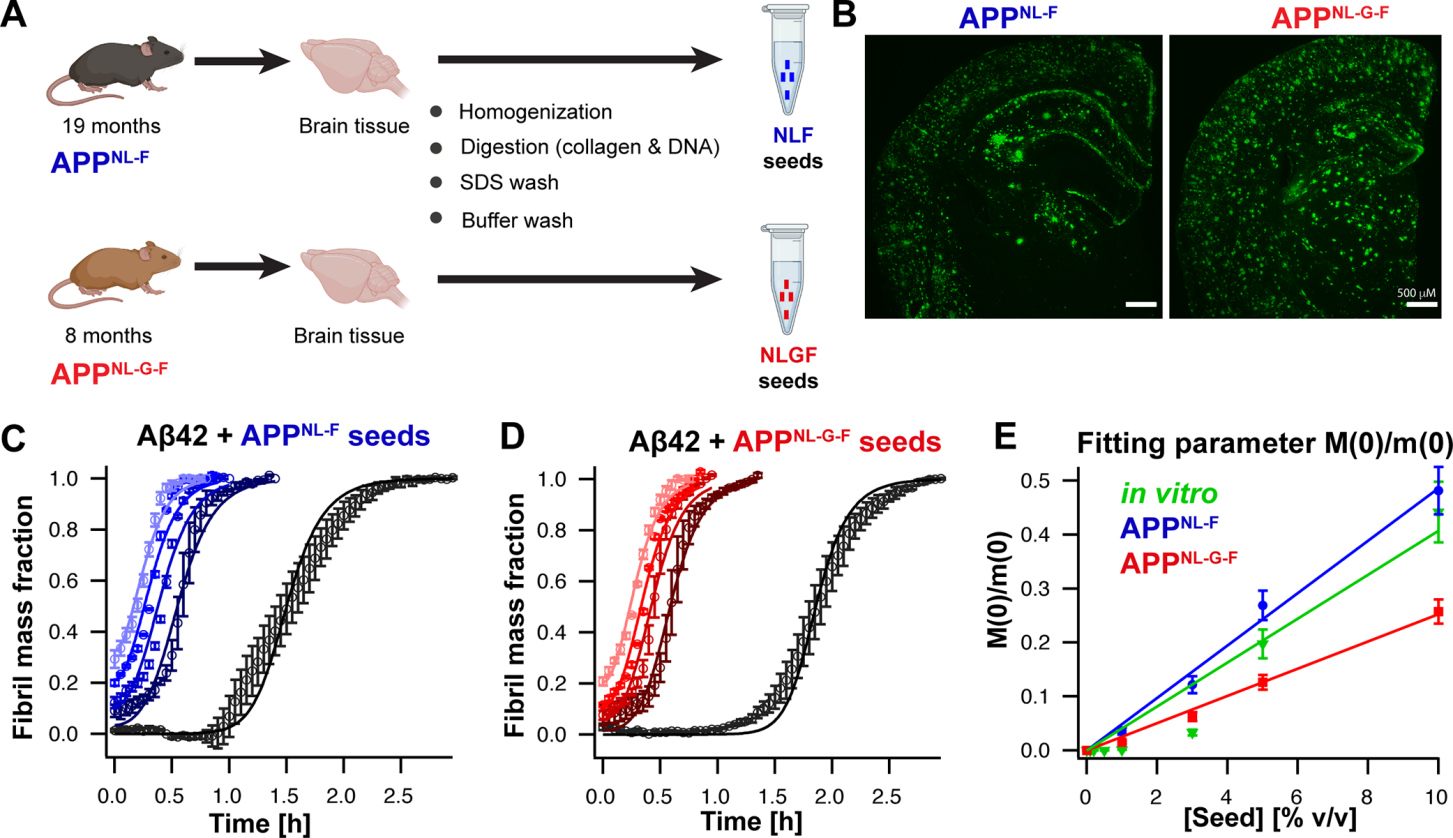
Aβ fibril extraction from App^NL-F^ and App^NL-G-F^ mouse brains and their seeding activity. (A) Schematic overview of Aβ fibril extraction protocol. (B) Representative images of immuno-stained brain sections of App^NL-F^ and App^NL-G-F^ mice using the 82E1 anti-Aβ antibody, revealing the presence of amyloid plaques. (C–E) Seeding activity of in vivo-derived fibrils from App^NL-F^ (C) and App^NL-G-F^ (D) mice using seed concentrations of 0, 1, 3, 5, and 10% v/v represented by dark to light colors from low to high seed concentration. The aggregation kinetics could be fitted globally to a nucleation model in which only the initial seed concentration, *M*(0), is the sole free-fitting parameter. The fitting parameter *M*(0) correlates linearly with the added seed concentration of the amyloid fibril extract (E), showing that the seeding activity of in vivo-derived fibrils can be described in a quantitative manner.

We extracted Aβ fibrils from the cerebrum of two different App knock-in AD mouse models using an established protocol (Figure 1A). We chose 19 month old App^NL-F^ and 8 month old App^NL-G-F^ mice since these mice exhibit abundant Aβ plaques in the brain.^12^ Immuno-stained images of brain sections confirmed abundant Aβ42 plaques in both App^NL-F^ and App^NL-G-F^ brain tissues (Figure 1B). The mouse brains were dissected,^24^ homogenized in Tris-calcium buffer, pH 8.0, and centrifuged, where the procedure was repeated three times in total (see Materials and Methods). After digestion with collagenase and DNAase I, the pellet was washed twice with a sodium dodecyl sulfate (SDS) buffer and washing buffer. Finally, the Aβ fibril-containing pellet was suspended in sodium phosphate buffer, pH 8.0.

The presence of Aβ in the mouse brain extract was visualized by western blot, which indeed confirmed that Aβ was present (Supporting Information Figure S1). Further, fibrillar morphology was observable for both App^NL-F^ and App^NL-G-F^ brain extracts in transmission electron microscopy (TEM) images (Supporting Information Figure S2). To validate that the observed fibrillar aggregates were formed from Aβ, we recorded immuno-electron microscopy (EM) micrographs of AD mouse brain extracts using a secondary antibody tagged with 5 nm gold nanoparticle. These immuno-EM images revealed the presence of gold nanoparticles on the surface of the fibrillar aggregates, suggesting that these fibrils indeed contain Aβ peptides. Notably, control experiments on brain extract from wild-type mice using the same protocol did not show any fibrillar aggregates on EM images (Supporting Information Figure S2). Furthermore, extracts from App^NL-G-F^ mouse cerebellum, where Aβ concentrations are lower than in cerebrum (Supporting Information Figure S1), did not display any fibril-like structures (Supporting Information Figure S2).

### Seeding Activity of In Vivo-Derived Fibrils from AD Mice

Having characterized the amyloid fibril extract, we asked whether extracted Aβ fibrils have the potential to seed Aβ42 monomers. To test this, we performed Thioflavin T (ThT) assays in the presence of sonicated in vivo-derived fibrils. Both App^NL-F^ and App^NL-G-F^ fibrils showed an overall similar seeding pattern, decreasing the aggregation lag time with increasing concentration of seeds (Figure 1C,D and Supporting Information Figure S3). Using the material obtained upon applying the same extraction protocol on wild-type mouse brains resulted in a concentration-dependent delay of Aβ42 aggregation kinetics, potentially due to the presence of additional inhibiting compounds (Supporting Information Figure S4). Similarly, extracts from the cerebellum from App^NL-F^ and App^NL-G-F^ delayed the Aβ42 aggregation kinetics (Supporting Information Figure S4).

To quantify the seeding activity of in vivo-derived fibrils, we applied a nucleation model including primary and secondary nucleation, with the reaction orders *n*_*C*_ and *n*_2_, respectively, in addition to fibril-end elongation. This model is dependent on the initial Aβ42 monomer concentration *m*(0), the initial fibril mass concentration *M*(0), and the fibril number (or polymer) concentration *P*(0). Notably, the fibril mass concentration *M*(0) and the polymer number concentration *P*(0) are linked by the average fibril length *L*, and to avoid overfitting of coupled fitting parameters the average fibril length was set to a constant value of *L* = 10 000.^25^ Further, the nucleation rate constants for primary (*k*_n_) and secondary (*k*_2_) nucleation as well as fibril elongation (*k*_+_) were assigned to global fitting parameters; i.e., they were constrained to the same value for all fibril concentrations. These constraints left *M*(0) as the only individual fitting parameter. The global fit analysis revealed good fits for both data sets from App^NL-F^ and App^NL-G-F^ mice (Figure 1C,D). Also, aggregation kinetic traces seeded with in vitro fibrils can be fitted with this model (Supporting Information Figure S5), as reported previously in the literature.^7^ Notably, in vitro seeded aggregation traces exhibit a considerably steeper slope compared to the nonseeded one, which is difficult to adjust by the current model and resulted in only a moderate fit of the unseeded kinetic trace (Supporting Information Figure S5). If *k*_2_ is applied as an additional free-fitting parameter this trace can be described well, pointing toward that, due to the high seeding capacity of in vitro seeds, the aggregation model needs to be adjusted to describe the whole seed concentration range. This analysis showed that seeding activity of in vivo-derived seeds can be described quantitatively, which is confirmed by the linear correlation between the amount of added seeds and the fitting parameter *M*(0), evident for both mouse models and in vitro seeds (Figure 1E). Interestingly, the App^NL-G-F^ extract, which contains Artic Aβ42 fibrils, can efficiently seed wild-type Aβ42, similarly as previously observed in vitro.^26^

### Morphology of In Vivo-Derived Aβ42 Fibrils

**Figure 2:**
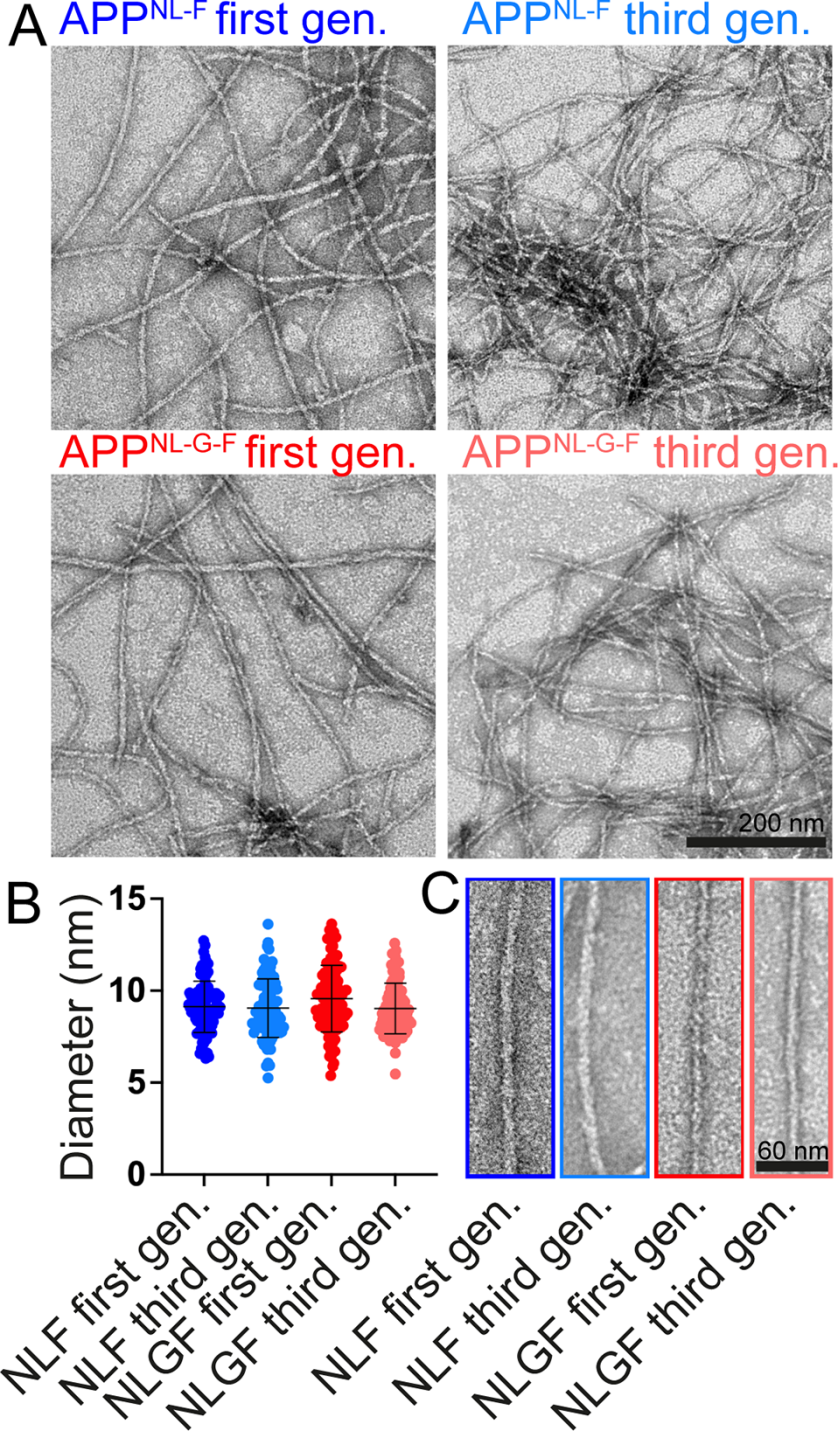
Morphology and structural characteristics of Aβ42 fibrils obtained with in vivo-derived seeds. (A) TEM images revealing the morphology of Aβ42 fibrils seeded with App^NL-F^ and App^NL-G-F^ extracts displayed for the first and third generations. (B) Scatterplot showing the diameters of first- and third-generation fibrils seeded with App^NL-F^ and App^NL-G-F^ brain extracts. (C) Enlarged images of first- and third-generation fibrils.

To shed light on the morphology of the in vivo-seeded fibrils, we analyzed the end products of the aggregation kinetics by TEM. We found characteristic fibrillar morphologies for both App^NL-F^ and App^NL-G-F^ seeded aggregation products (Figure 2A). Interestingly, the average diameter of fibrils was basically identical, with 9.1 ± 1.4 and 9.6 ± 1.8 nm for App^NL-F^ and App^NL-G-F^ seeded fibrils, respectively (Figure 2B), which coincide with the interval range of in vitro wild-type and Artic Aβ42 fibrils reported in previous studies.^27,28^ Notably, while in vitro fibrils typically exhibit a twist, which can be characterized by the node-to-node distance,^27,28^ the in vivo-derived fibrils obtained here appear rather straight, and no twist length could be determined.

Previous structural studies have used several generations of seeding to obtain a clearer fibril morphology,^29^ e.g., where fibril extracts from AD patient brain material were used as seeds for molecular structure determination of the seed-derived fibrils,^30^ and we applied the same strategy here. The fibrils of the first generation were sonicated and added to fresh monomeric Aβ42 to obtain the next generation of fibrils. Third-generation fibrils, obtained after performing the seeding procedure twice, exhibited average diameters of very similar values as for the first generation, with 9.1 ± 1.6 and 9.1 ± 1.4 nm for App^NL-F^ and App^NL-G-F^ seeded fibrils, respectively (Figure 2B). This suggests that the seeding with App^NL-F^ and App^NL-G-F^ extracts imprints the diameter of the in vivo fibril onto several generations of fibrils (Figure 2C).

### In Vivo-Derived Aβ42 Fibrils Exhibit Neurotoxic Effects in Electrophysiological Experiments

**Figure 3:**
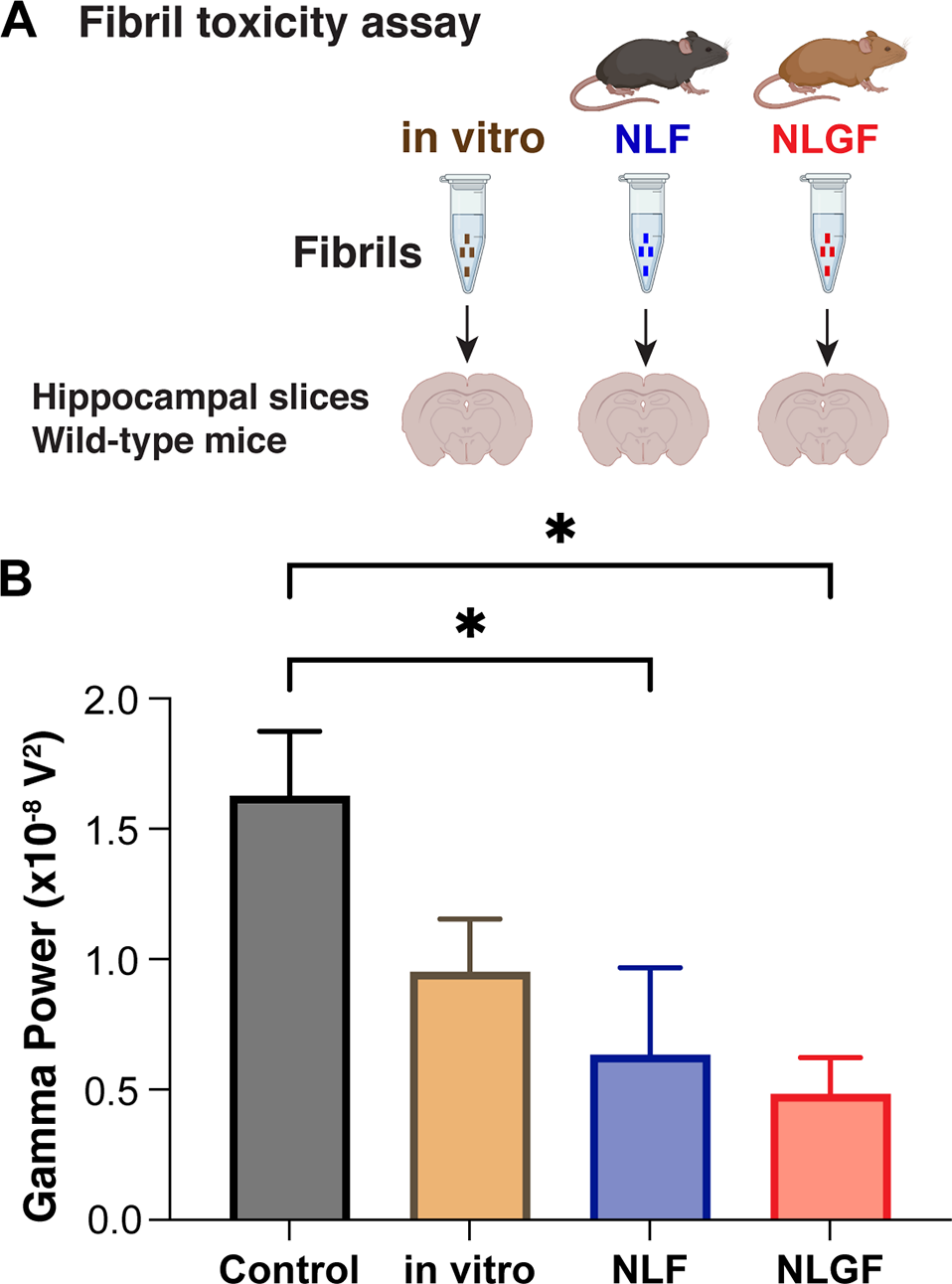
Effects of in vitro and in vivo-derived Aβ42 fibril-induced toxicity. (A) Schematic experimental setup where the toxicity is measured by the impact on γ-oscillations in mouse hippocampal slices ex vivo upon preincubation of 0.3 nM Aβ42 fibrils, in vitro and from App^NL-F^ and App^NL-G-F^ fibril extracts. (B) Fibril extracts from App^NL-F^ and App^NL-G-F^ mice exhibit significant toxic effects compared to control experiments.

To investigate the toxicity of in vivo-derived fibrils obtained from App^NL-F^ and App^NL-G-F^ mouse brains, we performed electrophysiological studies on mouse hippocampal slices ex vivo. The attenuation of γ-oscillations has previously been linked to cognitive decline in AD patients.^31−33^ Previous studies found neurotoxic effects of in vitro Aβ42 fibrils in electrophysiological studies,^34^ and small amounts of in vitro Aβ42 fibrils were shown to increase the toxic effects of recombinant Aβ42 monomers, presumably by enhancing secondary nucleation reactions, which promote the generation of neurotoxic species.^35^ Hippocampal slices were incubated with Aβ42 fibrils formed de novo or with fibrils directly derived from App^NL-F^ or App^NL-G-F^ brain extracts at a fibril concentration of 0.3 nM determined by western blot. Then, γ-oscillations in hippocampal slices of wild-type mice were induced by superfusing 100 nM kainite. All fibril types decreased the power of the γ-oscillations, where extracts from App^NL-F^ and App^NL-G-F^ exhibited significant attenuation compared with the control sample (Figure 3). The impact of in vivo-derived fibrils is seemingly larger than that for in vitro fibrils, although the difference between the different fibril types is not to a significant extent (Figure 3). These results hence suggest that in vivo-derived Aβ42 fibrils exhibit similar or even stronger neurotoxic effects than in vitro Aβ42 fibrils.

### Effect of Bri2 BRICHOS on App^NL-F^ and App^NL-G-F^ Seeded Aβ42 Aggregation Kinetics

The BRICHOS domain has previously been shown to efficiently delay Aβ42 and Aβ40 aggregation in vitro in a concentration-dependent manner.^22,23,35−37^ The rh Bri2 BRICHOS R221E monomer mutant, used in this study and termed BRICHOS, predominately inhibits secondary nucleation processes during Aβ42 self-assembly, in addition to a smaller effect on fibril-end elongation.^23^ To make conclusions about its mechanism of action in vivo, a crucial question is whether and how BRICHOS modulates the aggregation of Aβ42 seeded with in vivo-derived fibrils. Hence, we performed here aggregation kinetics assays of Aβ42 seeded with low (1% v/v of added seed extract) and high (10% v/v of added seed extract) concentrations of seeds from App^NL-F^ and App^NL-G-F^ mice in the presence of different molar equivalents of BRICHOS. In general, primary nucleation processes are negligible in the presence of low seed concentrations, and nucleation events connected to secondary nucleation and fibril-end elongation are dominant.^38^ Furthermore, the presence of a high seed concentration provides a large amount of free fibril ends, creating a condition where the start of the nucleation reaction is dominated by fibril-end elongation events. Hence, under this condition the elongation rate is proportional to the initial slope of the reaction profile.^38^

**Figure 4:**
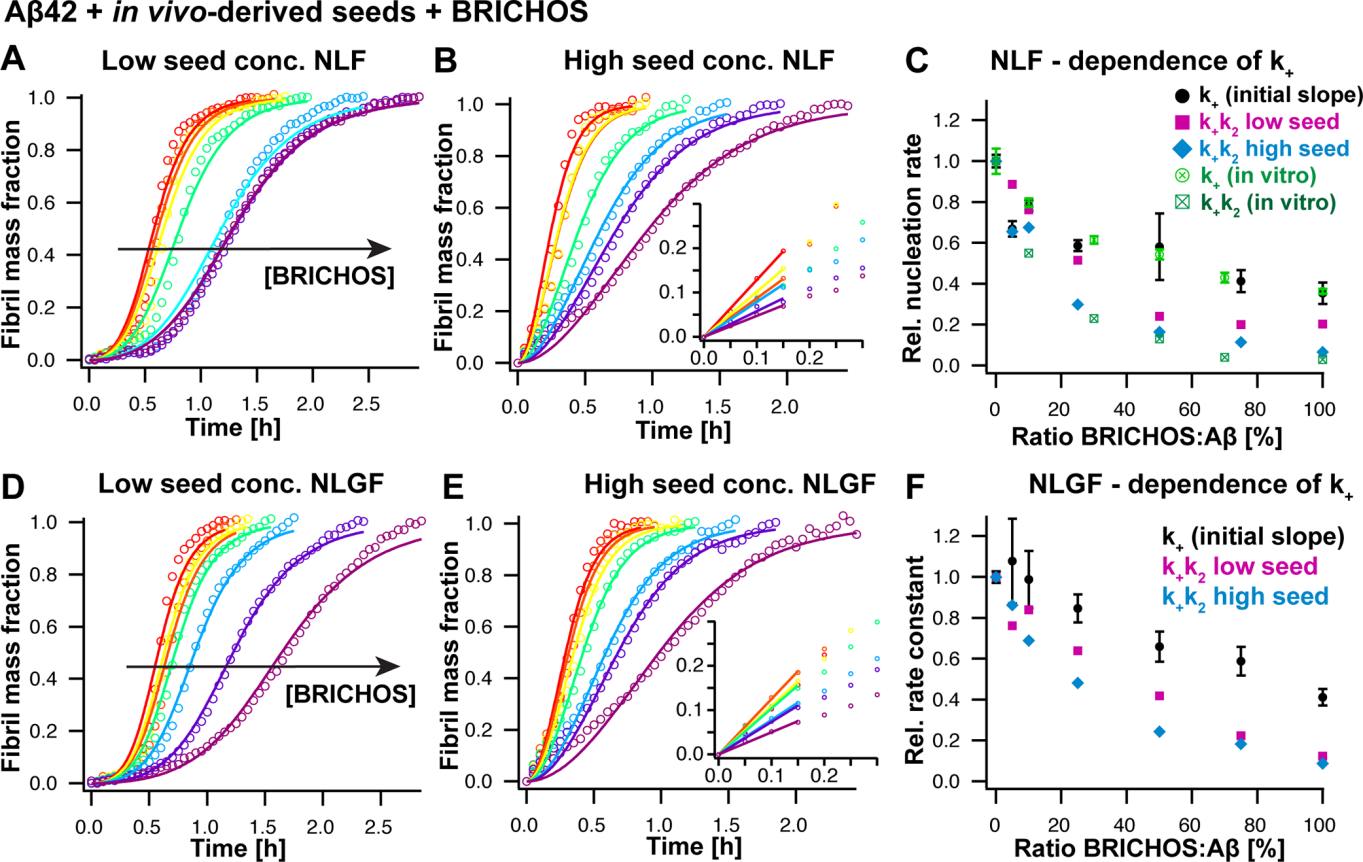
Inhibitory effect of BRICHOS on Aβ42 aggregation kinetics seeded by App^NL-F^ and App^NL-G-F^ in vivo-derived fibrils. (A, B, D, E) Aggregation kinetics at low (1% v/v of added seed extract) and high seed concentration (10% v/v of added seed extract) seeded by App^NL-F^ (A, B) and App^NL-G-F^ (D, E) in vivo-derived seeds in the presence of 0 (red), 5 (orange), 10 (yellow), 25 (green), 50 (turquoise), 75 (violet), and 100% (purple) molar equivalents of BRICHOS. (C, F) Dependence of the global fitting parameters *k*_+_ and *k*_2_ (shown as the combined rate constant *k*_+_*k*_2_) and the initial slope from highly seeded aggregation kinetics (corresponding to *k*_+_) on the molar ratio of Aβ/BRICHOS. In (C) previously determined parameters *k*_+_ and *k*_+_*k*_2_ from aggregation kinetics without the presence of in vivo-derived seeds are displayed in green color,^23^ showing that the dependence of the initial slope *k*_+_ and the global fitting parameter *k*_+_*k*_2_ exhibit a very similar behavior in the presence of in vivo-derived seeds.

We found that BRICHOS inhibits Aβ42 aggregation at low and high seed concentrations for both App^NL-F^ and App^NL-G-F^ in vivo-derived fibrils. The aggregation kinetics were fitted with the same nucleation model (Figure 4A,B and D,E) as that applied for the seeded aggregation kinetics without BRICHOS (Figure 1). Of notice, the fibril mass fractions in Figure 4 are plotted starting from zero, i.e., without the initial seed fibril mass, corresponding to the fibril mass fraction formed from initially monomeric Aβ42 peptides. A global fit, where only the nucleation rate constant *k*_2_ is a free individual fitting parameter, described very well the observed aggregation behavior both at low and at high seed concentrations (Figure 4A,B and D,E and Supporting Information Figure S6). Of importance, the elongation rate constant *k*_+_ and the secondary nucleation rate constant *k*_2_ are coupled parameters, and hence a fit where either *k*_+_ or *k*_2_ is the only individual fitting parameter resulted in identical fits (Supporting Information Figure S7). The dependence of these fitting parameters on the BRICHOS concentration is thus given as the combined nucleation rate constant (Figure 4C,F). On the contrary, a fit where only the primary nucleation rate constant *k*_n_ is the free fitting parameter cannot describe the aggregation traces (Supporting Information Figure S7).

At high seed concentration the initial slope was determined by a linear fit to the first data points (Figure 4B,E, insets). The relative initial slopes, representing the elongation rates, exhibit a weaker decrease with increasing concentrations of BRICHOS compared to the fitting parameter of the combined nucleation rate constants *k*_+_*k*_2_ (Figure 4C,F). In contrast, the combined nucleation rate constants from the data sets at low and high seed concentrations show a very similar dependence on the BRICHOS concentration. This indicates that a sole contribution of *k*_+_ is not sufficient to describe the observed behavior and instead a contribution of both *k*_+_ and *k*_2_ is required to fully capture the aggregation modulation.

Interestingly, the inhibitory effect of BRICHOS is very similar for both App^NL-F^ and App^NL-G-F^ in vivo-fibril-seeded aggregation kinetics. This suggests that the underlying molecular mechanisms of how BRICHOS interferes with the Aβ42 self-assembly is the same independently whether the aggregation is accelerated by App^NL-F^ and App^NL-G-F^ in vivo-derived fibrils.

## Conclusion

The findings presented here show that Aβ fibrils can be successfully isolated from App^NL-F^ and App^NL-G-F^ mouse brains, and the fibril extracts can potently seed Aβ42 aggregation. The seeding activity can quantitatively be described using a global fit analysis, revealing that these in vivo-derived fibrils accelerate Aβ42 self-assembly in a similar manner as previously found for in vitro Aβ42 fibrils.^7^ Further, the in vivo-derived fibrils from App^NL-F^ and App^NL-G-F^ mice share similar structural characteristics and exhibit comparable toxic effects, as measured by electrophysiological experiments. Moreover, we found that the BRICHOS protein efficiently inhibits Aβ42 fibrillization seeded with in vivo-derived fibrils by reducing the rates of secondary nucleation and fibril-end elongation, similarly as previously reported for in vitro Aβ42 aggregation.^23,36^

Taken together, these results suggest that in vivo-derived fibrils from disease-relevant mouse models exhibit seeding activities similar to those of in vitro Aβ42 fibrils, promoting surface-catalyzed secondary nucleation processes similar to the in vitro counterparts. Further, the inhibitory effect of BRICHOS, as an example of a molecular chaperone targeting amyloid generation, is transferable from in vitro to in vivo-derived fibril-seeded Aβ42 aggregation kinetics, pointing toward a similar mechanism of action of preventing Aβ42 self-assembly being present in vivo. This provides a molecular understanding of how protein-based treatments can attenuate neuroinflammation and improve cognitive behavior in AD mouse models,^24,42^ directing ways on how molecular chaperones can be utilized to combat toxic amyloid formation in neurodegenerative diseases.

## Materials and Methods

### Aβ42 and Bri2 BRICHOS Expression and Purification

Aβ42 and Bri2 BRICHOS were expressed and purified as previously reported^22,39^ where a detailed description can be found in Supporting Information.

### Aβ Fibril Extraction from AD Mouse Models

App^NL-F^ (∼19 months of age) and App^NL-G-F^ (∼8 months of age) mice were anesthetized with isoflurane 1.5%, followed by cardiac perfusion with phosphate-buffered saline (PBS). The mouse brain hemispheres were dissected into left and right brain hemispheres and stored at −80 °C until further use. For Aβ amyloid extraction, we used a modified protocol, which was recently published for extraction of Aβ fibrils from AD patients’ brains.^40^ Brain material was homogenized in 0.5 mL of Tris calcium buffer (20 mM Tris, 138 mM NaCl, 2 mM CaCl_2_, 0.1% NaN_3_, pH 8) in a microfuge tube. The homogenized solution was centrifuged at 21 000*g* for 30 min where the supernatant was discarded, and this process was repeated twice. The pellet obtained after the third homogenization step was then suspended in 1 mL of Tris-calcium buffer, and 10 μL of Dnase I (14.6 Kunitz, sigma) and 50 μL of collagenase (5 mg/mL) were added to it. This was then incubated overnight at 37 °C. On the following day, the solution was centrifuged at 21 000*g* for 30 min, after which we discarded the supernatant. The pellet was resuspended in 1 mL of washing buffer (50 mM Tris, 10 mM ethylenediaminetetraacetic acid (EDTA), pH 8), and 20 μL of 10% SDS was added to the solution. The solution was vortexed, incubated at 37 °C for 30 min, and centrifuged at 21 000*g* for 30 min. The pellet was dissolved again in washing buffer, and the above process was repeated with the addition of SDS. Finally, two more washes were done without SDS. The pellet obtained after the final washing step was suspended in 50 μL of 20 mM sodium phosphate buffer, 0.2 mM EDTA, pH 8.0. This fibril brain extract was used for further experiments. Nontransgenic (C57BL/6) mouse brains were similarly processed as control samples. Experiments were performed with ethical approvals for App^NL-F^ and App^NL-G-F^ mice from the Swedish “Linköping’s animal ethical board” under Dnr 03049-2020.

### ThT Fluorescence Assay for Aggregation Kinetics Measurements

The fluorescence experiments were recorded on 3 μM Aβ42 with a FLUOStar Galaxy (BMG Labtech) fluorimeter at 37 °C under quiescent condition. Aggregation kinetics was performed in 384-well plates with four replicates where each replicate contained 20 μL of sample in 20 mM sodium phosphate, pH 8, with 20 μM ThT. For the seeding with App^NL-F^ and App^NL-G-F^ mouse brain extracts, the amyloid extracts were sonicated (2 pulse on, 2 pulse off, 20% amplitude, for 3 min), and different seed percentage volumes of the fibril extract were added to a 3 μM Aβ42 monomer sample. For in vitro controls, six replicates were used, and in vitro fibril seeds (sonicated mature fibrils) were added in concentrations from 0.1 to 10%. To study the effect of Bri2 BRICHOS on App^NL-F^ and App^NL-G-F^ seeded Aβ42 aggregation, we used the lowest (1% v/v) and highest (10% v/v) volume of seeds with various molar ratios of BRICHOS with respect to 3 μM Aβ42.

For different seeding generations, 5% v/v seed extract was added to 3 μM Aβ42 monomer, which corresponds to first-generation seeds. Ten percent (v/v) seeds of the original sample was added to 3 μM Aβ42 monomer and incubated for 24 h, generating the next generation of fibril.

### Global Fitting Analysis

Individual aggregation traces were normalized, and the median over four replicates was used for further analysis. For the normalization of seeded aggregation kinetics, the ratio of the initial intensity to the final intensity was maintained, subtracting the baseline of unseeded kinetics. Aggregation traces were truncated at the plateau of the aggregation reaction, which exhibits approximately the same baseline-subtracted values as the traces without seeds (Supporting Information Figure S3) or without BRICHOS (Supporting Information Figure S6). The aggregation kinetics at different added seed concentrations was fitted globally by applying a nucleation model, which includes primary and secondary nucleation in addition to fibril-end elongation. The time dependence of the fibril mass fraction is given by^7^
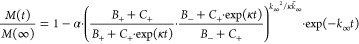
1where the additional coefficients are functions of the microscopic rate constants *k*_n_, *k*_+_, *k*_2_, the initial monomer concentration *m*(0), the initial seed concentration *M*(0), and the initial polymer concentration *P*(0). The reaction orders *n*_C_ and *n*_2_ for primary and secondary nucleation, respectively, were set to *n*_C_ = *n*_2_ = 2, as determined previously for Aβ42 aggregation.^3^
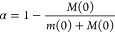

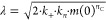

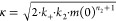

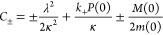

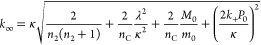

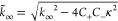

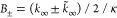


The initial polymer *P*(0) concentration and seed concentration are related by the average fibril length *L* = *M*(0)/P(0). To avoid overfitting, an average fibril length *L* = 10 000 was assumed for all seed concentrations.

For aggregation kinetics with different seed concentrations, the nucleation rate constants *k*_n_, *k*_+_, and *k*_2_ were set as global fit parameters, leaving the initial seed concentration *M*(0) as the only free individual fitting parameter.

For aggregation kinetics using a fixed seed concentration and different BRICHOS concentrations, the aggregation traces were normalized subtracting the initial fibril mass concentration; i.e., this normalization reflects the fibril mass generated from initially monomeric recombinant Aβ42 peptides. For the fitting, the normalization parameter α in eq 1 is set to one. The rate constants *k*_+_ and *k*_2_ are coupled and, hence, indistinguishable. For the global fits, *k*_n_ and *k*_+_ were set to constant values, as well as the initial seed concentration *M*(0), as obtained from the previous analysis with different seed concentrations. These constraints leave the nucleation rate constant *k*_2_ as the only free individual fitting parameter, representing the coupled parameters *k*_+_ and *k*_2_.

### Hippocampal Slice Preparations for Electrophysiology Measurements

Wild-type (WT) mice were used to test the effect of Aβ fibrils extracted from brain homogenates of App^NL-F^ and App^NL-G-F^ mice on WT hippocampal γ-oscillations. γ-Oscillations were measured for 0.3 nM in vitro Aβ42 fibrils and 0.3 nM Aβ fibrils from APP^NL-F^ and APP^NL-G-F^, which were directly extracted from brain samples and applied without any amplification by seeding.^23,35,41^ A detailed description is given in the Supporting Information. Experiments were conducted with the ethical approval by the Swedish “Norra Stockholm’s Djurförsöksetiska Nämnd” with Dnr N45/13.

### TEM and Immuno-EM Analysis

TEM imaging was performed using an FEI Tecnai 12 Spirit BioTWIN, operated at 100 kV with a 2 × 2 k Veleta CCD camera (Olympus Soft Imaging Solutions, GmbH, Münster, Germany). 10–15 images were taken randomly for each sample at different magnifications between 20 000× and 60 000×. Five microliters of sample was spotted on a 400 mesh Formvar/carbon-coated copper grid and incubated for 10 min. The grid was then washed twice with 5 μL of Milli-Q (MQ) water, stained with 5 μL of 1% uranyl formate for 5 min, and air-dried. For immuno-EM, 5 μL samples were spotted on the 200-mesh nickel grid. After 10 min, the excess sample was blotted with Whatman filter paper. The grid was blocked by using 5 μL of 1% bovine serum albumin (prepared in PBS). After 30 min, the grid was washed thrice with MQ water. Then, 10 μL of primary mouse antibody (6E10, 1:200 in PBST, BioLegend) was dropped on the grid and incubated at room temperature for 60 min. It was then washed thrice with PBST followed by incubation with secondary antibody (antimouse IgG-gold, 1:40 dilution in PBST, BBI Solutions, Crumlin, UK) for 60 min. The grid was then washed three times with PBST and then stained as described above.

## Supporting Information Available

An additional methods and material description and figures (PDF)

## Abbreviations

Aβ: amyloid-β
AD: Alzheimer’s disease
App: amyloid precursor protein
HSP: heat shock protein
EM: electron microscopy
ThT: Thioflavin T
