# Supplementary material for: Molecular Mechanisms of Amyloid-β Self-Assembly Seeded by In Vivo-Derived Fibrils and Inhibitory Effects of the BRICHOS Chaperone
Source: ACS Chem Neurosci. 2023 Apr 6;14(8):1503–11. doi: 10.1021/acschemneuro.3c00044 (PMC10119923; doi:10.1021/acschemneuro.3c00044)
Supplement: Supplementary file 1 — cn3c00044_si_001.pdf [file cn3c00044_si_001.pdf]

Supporting Information

Molecular Mechanisms of Amyloid-β Self-assembly Seeded by *In Vivo*-Derived Fibrils and Inhibitory Effects of the BRICHOS Chaperone

Rakesh Kumar[1], Luis Enrique Arroyo-García[2,3], Shaffi Manchanda[1], Laurène Adam[1], Giusy Pizzirusso[2,3], Henrik Biverstål[1], Per Nilsson[2], André Fisahn[2], Jan Johansson[1] and Axel Abelein[1*]

[1] Department of Biosciences and Nutrition, Karolinska Institutet, 141 52 Huddinge, Sweden

[2] Division of Neurogeriatrics; Center for Alzheimer Research; Department of Neurobiology, Care Sciences and Society, Karolinska Institutet, 171 64 Solna, Sweden

[3] *Department of Women's and Children's Health, Karolinska Institutet, Solna, Sweden*

MATERIAL AND METHODS

Aβ42 and Bri2 BRICHOS expression and purification

Aβ42 was expressed and purified as previously reported.[1] Briefly, Aβ42 was expressed as fusion protein NT^*_{FlSp}-Aβ42 in *E. coli,* BL21(DE3). The protein expression was induced by IPTG addition in bacterial culture flask, when OD_{600} of cells reached ~0.8. The cells were pellet by centrifuging at 7000×g for 20 min. Then, the bacterial pellet was suspended in 8 M urea in 20 mM Tris-HCl, pH 8. Bacterial cells were then sonicated and centrifuged at 24000×g for 30 min. The supernatant was loaded on HisPrep™ FF 16/10 column and the protein was eluted with 300 mM imidazole, 8 M urea in 20 mM Tris-HCl. The fusion protein was dialyzed overnight in 4 °C and it was cleaved overnight with TEV protease (1:30 enzyme to substrate, w/w) with 1 mM Dithiothreitol (DTT) and 0.5 mM EDTA. Cleaved protein was lyophilized and dissolved in 10 ml of 7 M guanidine-HCl. The protein was further purified by size exclusion chromatography using Superdex 30, 26/600 (Cytiva) column. Monomeric Aβ42 was collected and the peptide absorbance was measured using UV spectrometer. From absorbance, the peptide concentration was calculated using extinction coefficient of Aβ42 (1424 M^{-1} cm^{-1}, A280-A300).

Bri2 BRICHOS R221E was expressed and purified as reported previously.[2] In brief, NT^*_{MaSp}-Bri2 BRICHOS was expressed in SHuffle T7 competent *E. coli* (K12 strain) cells. The cells were grown at 30 °C until the OD_{600} of cells reached ~0.8 and then the protein induction was done with 0.5 mM IPTG. Cells were grown further overnight at 20 °C. Next day, the cells were pelleted by centrifugation (7000×g for 20 min). Then, the cells were resuspended in 20 mM Tris-HCl, pH 8, sonicated and centrifuged at 24000×g for 30 min. The supernatant was loaded on a HisPrep™ FF 16/10 column using the ÄKTA system. The fusion Bri2 BRICHOS protein was eluted using 300 mM imidazole. The protein was dialyzed overnight using 10 kDa cut-off dialysis membrane. Then, the protein was cleaved overnight with Thrombin (1:1000). It was further purified with reverse IMAC where the flow-through was collected. The flow-through was injected in a size exclusion chromatography column (Superdex 75, 26/600), where monomeric Bri2 BRICHOS was collected and stored at −80 °C until further use.

Western and dot blots

The fibrils extracted from the AD mouse brains were dissolved in 50 % formic acid and then vortexed and incubated at 22 °C for 60 min. The samples were lyophilized and dissolved in 20 mM sodium phosphate buffer. A SDS-PAGE was performed to separate different proteins on a 4-20 % precast gel (Bio-Rad). Proteins were transferred on the nitrocellulose membrane for further process. For dot blot, samples were spotted directly on nitrocellulose membrane and air-dried. The further process is the same for western and dot blot. First, the membrane was blocked with 5 % milk for 30 min and then washed thrice with phosphate buffer saline, 0.1 % Tween (PBST). The membrane was then incubated with primary mouse antibody (6E10, 1: 5000 in PBST, BioLegend) at 4 °C overnight or 60 min at 22 °C. The membrane was then washed thrice with PBST and then incubated with HRP conjugated secondary anti mouse-antibody (1:5000 in PBST) for 60 min. Finally, the membrane was washed thrice with PBST and membrane was developed after addition of enhanced luminol-based chemiluminescent substrate (ECL prime, Cytiva). The imaging was conducted using an Amersham™ Imager 600.

Hippocampal slice preparations for electrophysiology measurements

We used wild type (WT) mice (n=16) at 20-30 postnatal days to test the effect of Aβ fibrils extracted from brain homogenates of App^{NL-F} and App^{NL-G-F} mice on WT hippocampal gamma oscillations. For brain extraction, mice were deeply anesthetized with isoflurane. The brain was dissected out and placed in ice-cold artificial cerebrospinal fluid (ACSF) modified for dissection containing (in mM): 80 NaCl, 24 $NaHCO_3$, 25 glucose, 1.25 NaH_2PO_4, 1 ascorbic acid, 3 Na-pyruvate, 2.5 KCl, 4 $MgCl_2$, 0.5 CaCl2, 75 sucrose and bubbled with carbogen (95% O2 and 5% CO2). Horizontal sections (350 μm thick) of the ventral hippocampi of both hemispheres were prepared with a Leica VT1200S vibratome (Leica Microsystems). Immediately after cutting, slices were transferred into a humidified interface holding chamber containing standard ACSF (in mM): 124 NaCl, 30 $NaHCO_3$, 10 glucose, 1.25 NaH_2PO_4, 3.5 KCl, 1.5 MgCl2, 1.5 $CaCl_2$, continuously supplied with humidified carbogen. The chamber was held at 34 °C during slicing and subsequently allowed to cool down to room temperature (~22 °C) for a minimum of 1 hour. Then, slices were transferred to an incubation chamber for 30 min, containing ACSF, ACSF + 0.3 nM *in vitro* Aβ42 fibrils, or ACSF + 0.3 nM Aβ fibrils from APP^{NL-F} and APP^{NL-G-F}.[3-4] After incubation time the slices were transferred to the interface-style recording chamber for extracellular recordings. During the incubation, slices were supplied continuously with carbogen gas (5% CO2, 95% O2) bubbled into the ACSF.

Recordings were performed with borosilicate glass microelectrodes filled with ACSF in hippocampal area CA3, pulled to a resistance of 3–6 MΩ. Local field potentials (LFP) were recorded at 32 °C in an interface-type chamber (perfusion rate 4.5 mL per minute). LFP gamma oscillations were elicited by kainic acid (100 nM). The oscillations were stabilized for 20 min before any recordings. Interface chamber LFP recordings were carried out by a 4-channel amplifier/signal conditioner M102 amplifier (Electronics lab, University of Cologne, Germany). The signals were sampled at 10 kHz, conditioned using a Hum Bug 50 Hz noise eliminator (Quest Scientific, North Vancouver, BC, Canada), software low-pass filtered at 1 kHz, digitized, and stored using a Digidata 1322 A and Clampex 10.4 programs (Molecular Devices, CA, USA). Power spectra density plots (from 60 s long LFP recordings) were calculated in averaged Fourier-segments of 8192 points using Axograph X (Kagi, Berkeley, CA, USA). Gamma oscillations power was calculated by integrating the power spectral density between 20 and 80 Hz with the result representing average values taken over 1 min periods.[5]

Experiments were conducted with the ethical approval by the Swedish “Norra Stockholm’s Djurförsöksetiska Nämnd” with Dnr N45/13.

SUPPORTING INFORMATION FIGURES

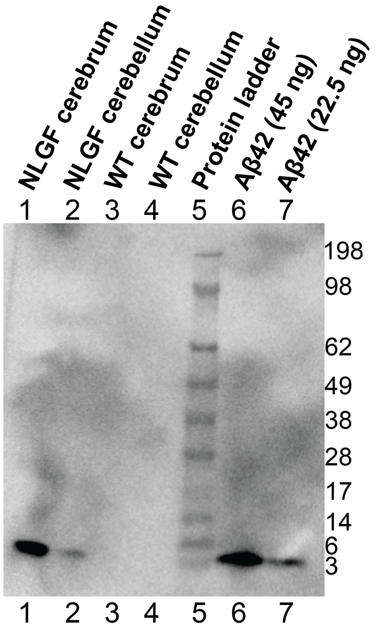

SI Figure S1: Western blot of fibril extracts. $APP^{NL\text{-}G\text{-}F}$ fibril extract of cerebrum (lane 1) and of cerebellum (lane 2) and corresponding amounts of non-tg WT brain from cerebrum (lane 3) and cerebellum (lane 4). Lane 5 shows the protein molecular ladder. Lane 6 and 7 represent monomeric Aβ42 with 45 ng and 22.5 ng for lane 6 and 7, respectively. These results show that Aβ is present in the $APP^{NL\text{-}G\text{-}F}$ brain extracts but not detectable in non-tg WT brain.

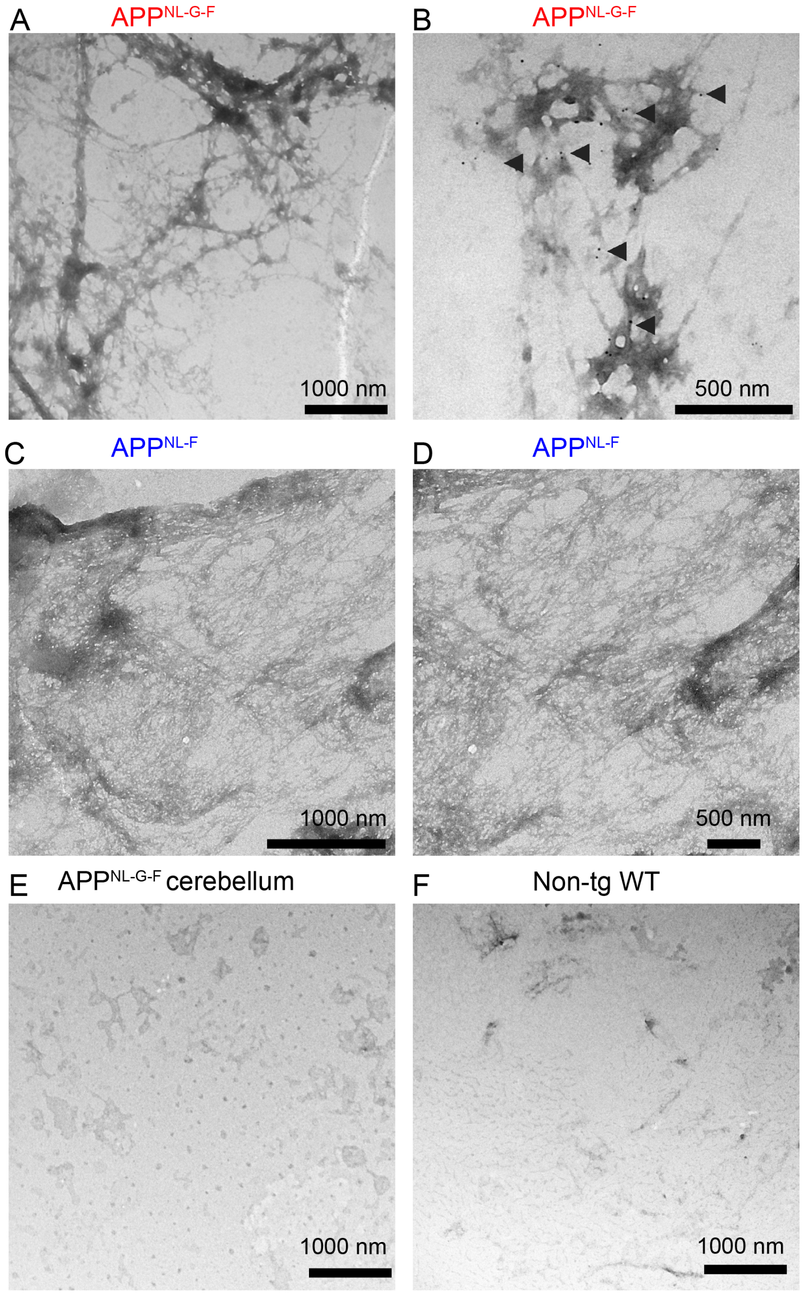

SI Figure S2: TEM images of *in vivo* fibrils directly extracted from the mouse brain. (A,B) EM (A) and immuno-EM (B) micrographs show fibril-like morphology for extracts from APP^{NL-G-F} brain tissue. The arrows in (B) point to examples of immunogold labeled fibrils. **(C,D)** EM images for APP^{NL-F} brain extract displays abundant fibril amounts. **(E,F)** EM images of the corresponding extracts from APP^{NL-G-F} cerebellum (E) and non-transgenic (non-tg) WT mouse cerebrum (F) show no detectable fibril-like aggregates.

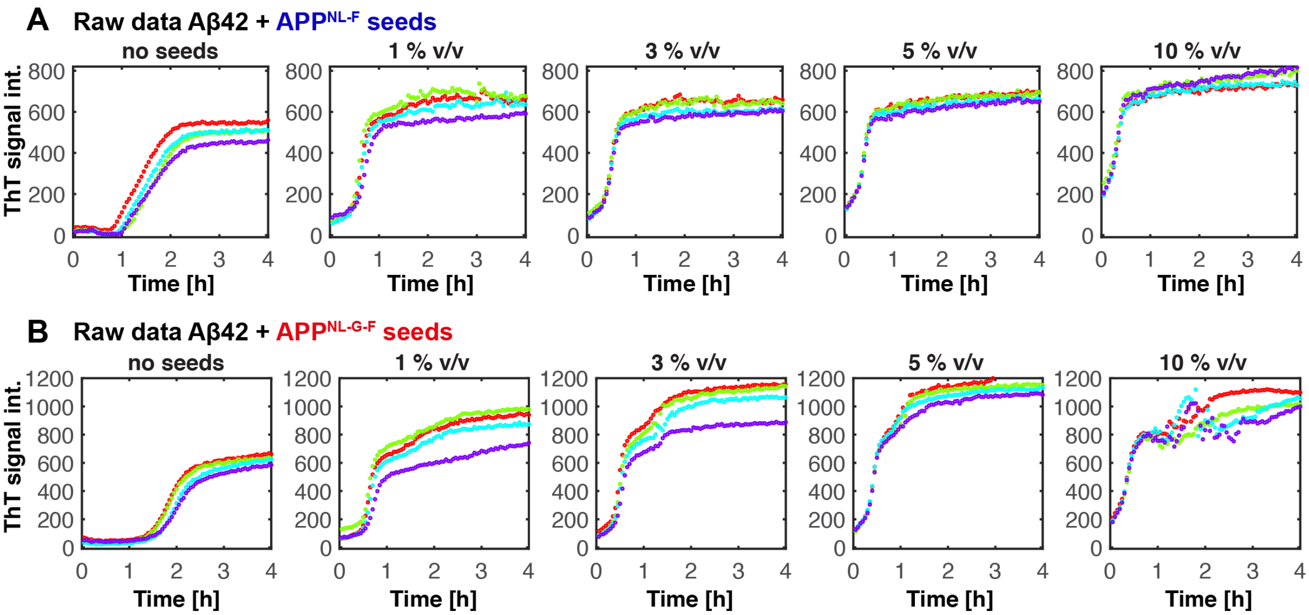

SI Figure S3: Raw data of Aβ42 aggregation kinetics in the presence of APP^{NL-F} & APP^{NL-G-F} derived fibrils. (A,B) Aggregation kinetics of Aβ42 in the presence of 0, 1, 3, 5 and 10 % v/v seeds derived from APP^{NL-F} (A) and APP^{NL-G-F} (B) brain extracts. The samples were run with four replicates.

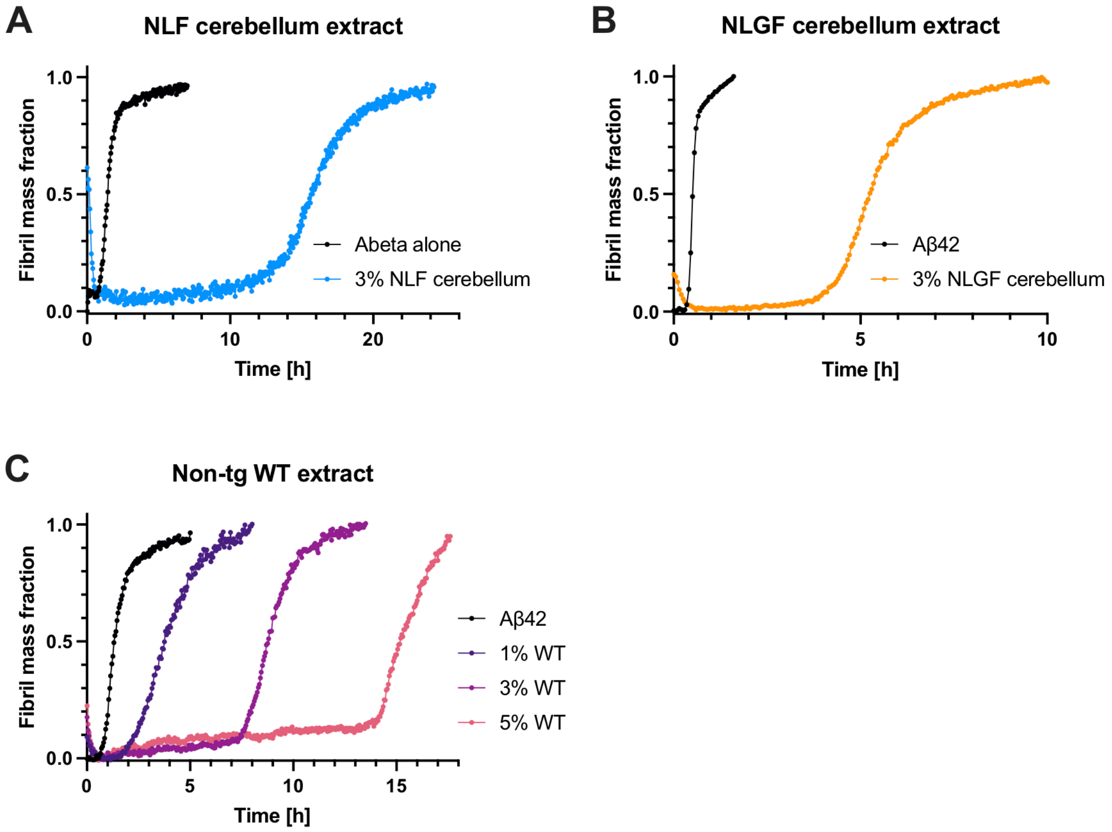

SI Figure S4: Aβ42 aggregation kinetics in the presence of extracts from APP^{NL-F} & APP^{NL-G-F} cerebellum and non-tg WT cerebrum. (A,B) Aggregation kinetics of 3 µM Aβ42 (black) in the presence of 3% v/v APP^{NL-F} (A, light blue) or APP^{NL-G-F} cerebellum (B, orange) or in the presence of 1% v/v (violet), 3% v/v (purple) and 5% v/v (light red) extract from non-tg WT cerebrum (C), showing a concentration-dependent delay of Aβ42 aggregation.

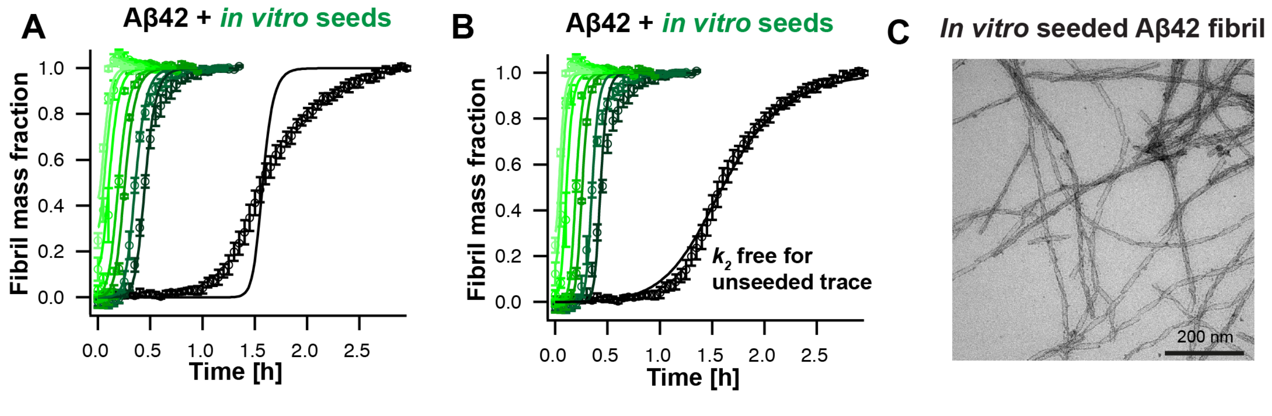

SI Figure S5: Aβ42 aggregation kinetics in the presence *in vitro* Aβ42 seeds. (A,B) Aggregation kinetics of Aβ42 in the presence of 0.1, 0.2, 0.5, 1, 2, 5, 10 % *in vitro* seeds represented by dark to light colors. While in (A) *M(0)* is the only free fitting parameter, resulting in only a moderate fit for the unseeded aggregation trace, in (B) k_2 is an additional free fitting parameter for the unseeded kinetic trace. (C) EM image of *in vitro* seeded Aβ42 fibrils.

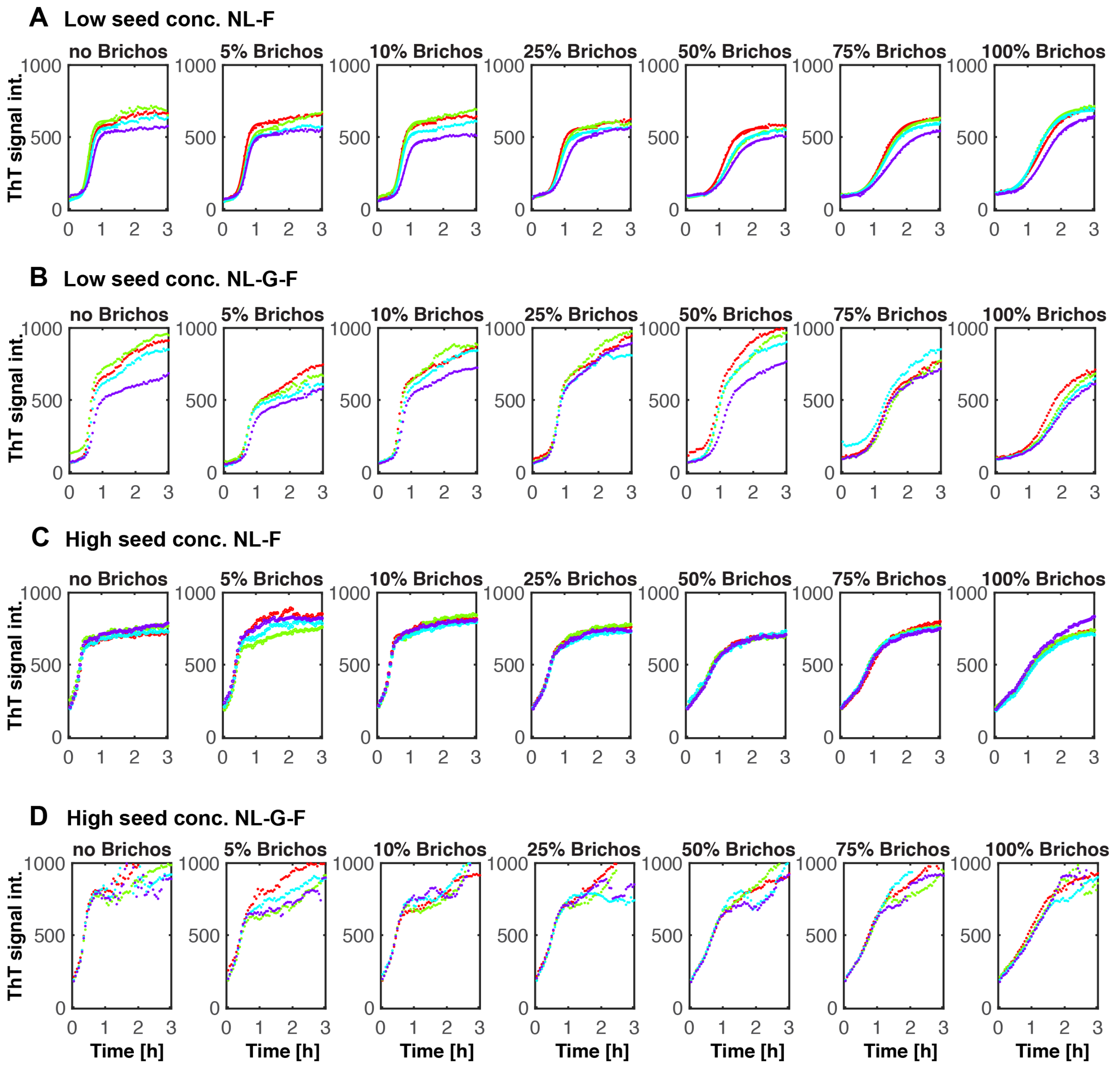

SI Figure S6: Raw data of Aβ42 aggregation kinetics in the presence *in vivo*-derived fibrils from $APP^{NL\text{-}F}$ & $APP^{NL\text{-}G\text{-}F}$ and different BRICHOS concentrations. (A-D) Aggregation kinetics of Aβ42 in the presence of 1 % v/v (A,B) and 10 % v/v seeds derived from $APP^{NL\text{-}F}$ (A,C) and $APP^{NL\text{-}G\text{-}F}$ (B,D) brain extracts in the presence of 0 to 100 % molar equivalents of BRICHOS. The samples were run with four replicates.

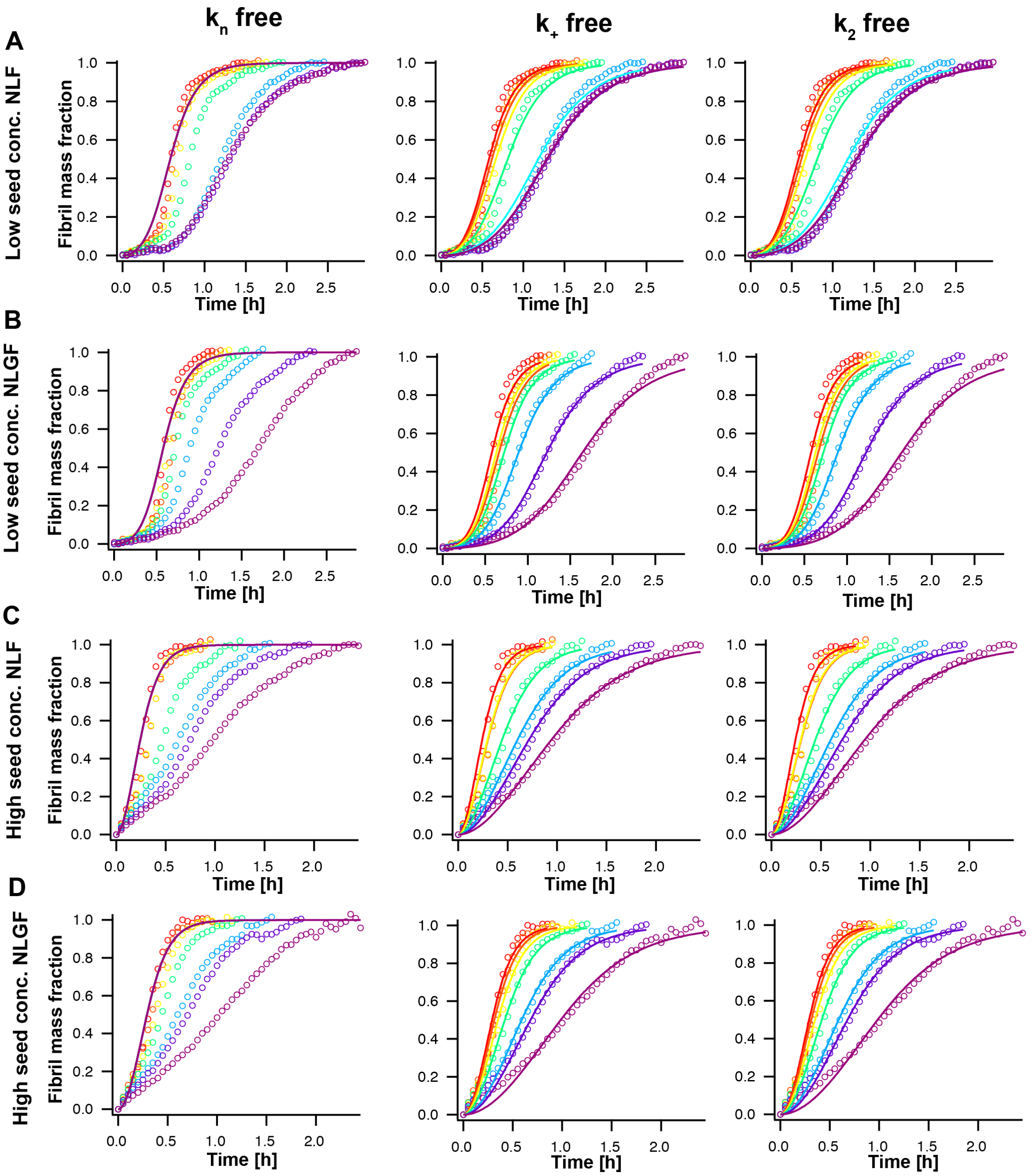

SI Figure S7: Global fits with one nucleation rate constant as free fitting parameter. Global fits of the aggregation kinetics in the presence of low (1% v/v) (A,B) and high (10% v/v) (C,D) seed concentrations from APP^{NL-F} (abbreviated as NLF) & APP^{NL-G-F} (abbreviated as NLGF) fibril extracts in the presence of 0 (red), 5 (orange), 10 (yellow), 25 (green), 50 (turquoise), 75 (violet) and 100% (purple) molar equivalents of BRICHOS. The analysis revealed that k_n cannot describe the aggregation kinetics as a free fitting parameter, while k_+ and k_2 result in equally good fits. The fits were additionally constrained to primarily fit the aggregation traces without BRICHOS to test the modulation effect at different BRICHOS concentrations. The poor fits with k_n as a free fitting parameter originates from $\lambda^2/\kappa^2 \ll M(0)/m(0)$ under the current conditions, resulting in that the seed concentration $M(0)$ constrains the global fit such that it only can represent the kinetic trace without BRICHOS. Since k_+ and k_2 are coupled parameters the fits with them as free fitting parameters are identical.

REFERENCES

1. Abelein, A.; Chen, G.; Kitoka, K.; Aleksis, R.; Oleskovs, F.; Sarr, M.; Landreh, M.; Pahnke, J.; Nordling, K.; Kronqvist, N.; Jaudzems, K.; Rising, A.; Johansson, J.; Biverstal, H., High-yield Production of Amyloid-beta Peptide Enabled by a Customized Spider Silk Domain. *Sci Rep* **2020,** *10* (1), 235.
2. Chen, G.; Abelein, A.; Nilsson, H. E.; Leppert, A.; Andrade-Talavera, Y.; Tambaro, S.; Hemmingsson, L.; Roshan, F.; Landreh, M.; Biverstal, H.; Koeck, P. J. B.; Presto, J.; Hebert, H.; Fisahn, A.; Johansson, J., Bri2 BRICHOS client specificity and chaperone activity are governed by assembly state. *Nat. Commun.* **2017,** *8* (1), 2081-017-02056-4.
3. Cohen, S. I. A.; Arosio, P.; Presto, J.; Kurudenkandy, F. R.; Biverstal, H.; Dolfe, L.; Dunning, C.; Yang, X.; Frohm, B.; Vendruscolo, M.; Johansson, J.; Dobson, C. M.; Fisahn, A.; Knowles, T. P.; Linse, S., A molecular chaperone breaks the catalytic cycle that generates toxic Abeta oligomers. *Nat. Struct. Mol. Biol.* **2015,** *22* (3), 207-213.
4. Chen, G.; Andrade-Talavera, Y.; Tambaro, S.; Leppert, A.; Nilsson, H. E.; Zhong, X.; Landreh, M.; Nilsson, P.; Hebert, H.; Biverstal, H.; Fisahn, A.; Abelein, A.; Johansson, J., Augmentation of Bri2 molecular chaperone activity against amyloid-beta reduces neurotoxicity in mouse hippocampus in vitro. *Commun Biol* **2020,** *3* (1), 32.
5. Arroyo-Garcia, L. E.; Isla, A. G.; Andrade-Talavera, Y.; Balleza-Tapia, H.; Loera-Valencia, R.; Alvarez-Jimenez, L.; Pizzirusso, G.; Tambaro, S.; Nilsson, P.; Fisahn, A., Impaired spike-gamma coupling of area CA3 fast-spiking interneurons as the earliest functional impairment in the App(NL-G-F) mouse model of Alzheimer's disease. *Mol. Psychiatry* **2021,** *26* (10), 5557-5567.
